# Microbial production of next-generation stevia sweeteners

**DOI:** 10.1186/s12934-016-0609-1

**Published:** 2016-12-07

**Authors:** Kim Olsson, Simon Carlsen, Angelika Semmler, Ernesto Simón, Michael Dalgaard Mikkelsen, Birger Lindberg Møller

**Affiliations:** 1 Evolva A/S, Lersø Park Alle 42-44, 5th, 2100 Copenhagen OE, Denmark; 2 Plant Biochemistry Laboratory, Department of Plant and Environmental Sciences, University of Copenhagen, Thorvaldsensvej 40, 1871 Frederiksberg C, Copenhagen, Denmark; 3 Center for Synthetic Biology “bioSYNergy”, University of Copenhagen, Thorvaldsensvej 40, 1871 Frederiksberg C, Copenhagen Denmark

## Abstract

**Background:**

The glucosyltransferase UGT76G1 from *Stevia rebaudiana* is a chameleon enzyme in the targeted biosynthesis of the next-generation premium stevia sweeteners, rebaudioside D (Reb D) and rebaudioside M (Reb M). These steviol glucosides carry five and six glucose units, respectively, and have low sweetness thresholds, high maximum sweet intensities and exhibit a greatly reduced lingering bitter taste compared to stevioside and rebaudioside A, the most abundant steviol glucosides in the leaves of *Stevia rebaudiana*.

**Results:**

In the metabolic glycosylation grid leading to production of Reb D and Reb M, UGT76G1 was found to catalyze eight different reactions all involving 1,3-glucosylation of steviol *C*_13_- and *C*_19_-bound glucoses. Four of these reactions lead to Reb D and Reb M while the other four result in formation of side-products unwanted for production. In this work, side-product formation was reduced by targeted optimization of UGT76G1 towards 1,3 glucosylation of steviol glucosides that are already 1,2-diglucosylated. The optimization of UGT76G1 was based on homology modelling, which enabled identification of key target amino acids present in the substrate-binding pocket. These residues were then subjected to site-saturation mutagenesis and a mutant library containing a total of 1748 UGT76G1 variants was screened for increased accumulation of Reb D or M, as well as for decreased accumulation of side-products. This screen was performed in a *Saccharomyces cerevisiae* strain expressing all enzymes in the rebaudioside biosynthesis pathway except for UGT76G1.

**Conclusions:**

Screening of the mutant library identified mutations with positive impact on the accumulation of Reb D and Reb M. The effect of the introduced mutations on other reactions in the metabolic grid was characterized. This screen made it possible to identify variants, such as UGT76G1_Thr146Gly_ and UGT76G1_His155Leu_, which diminished accumulation of unwanted side-products and gave increased specific accumulation of the desired Reb D or Reb M sweeteners. This improvement in a key enzyme of the Stevia sweetener biosynthesis pathway represents a significant step towards the commercial production of next-generation stevia sweeteners.

**Electronic supplementary material:**

The online version of this article (doi:10.1186/s12934-016-0609-1) contains supplementary material, which is available to authorized users.

## Background

The metabolic syndrome, which has become so prevalent in the industrialized countries throughout the world is associated with abdominal obesity, increased risks of developing cardiovascular diseases, and type 2 diabetes. This syndrome may be tracked back to our human ancestors who to meet their energy demand were dependent on carbohydrate rich cereals and ripe fruits that were recognized by their sweet taste. This innate attraction to sweet taste has become a severe health issue when food is plentiful and the diet’s calorie intake of sucrose and fructose syrups becomes excessive [1, 2].

To reduce the contribution of sucrose and other high-energy sweeteners to excessive human calorie intake, especially in the industrialized part of the world, chemically synthesized low- and zero-calorie sweeteners like saccharin and aspartame have been introduced, which 90% of American adults are using at least once every 2 weeks [3]. These have organoleptic drawbacks compared to sucrose and fructose syrups, and consumer demand for natural non-caloric sweeteners instead of chemically synthesized artificial sweeteners spurred a search for natural and generally healthy non-nutritive sweeteners. Steviol glucosides from the leaves of *Stevia rebaudiana* constitute such a natural alternative [4].

**Fig. 1:**
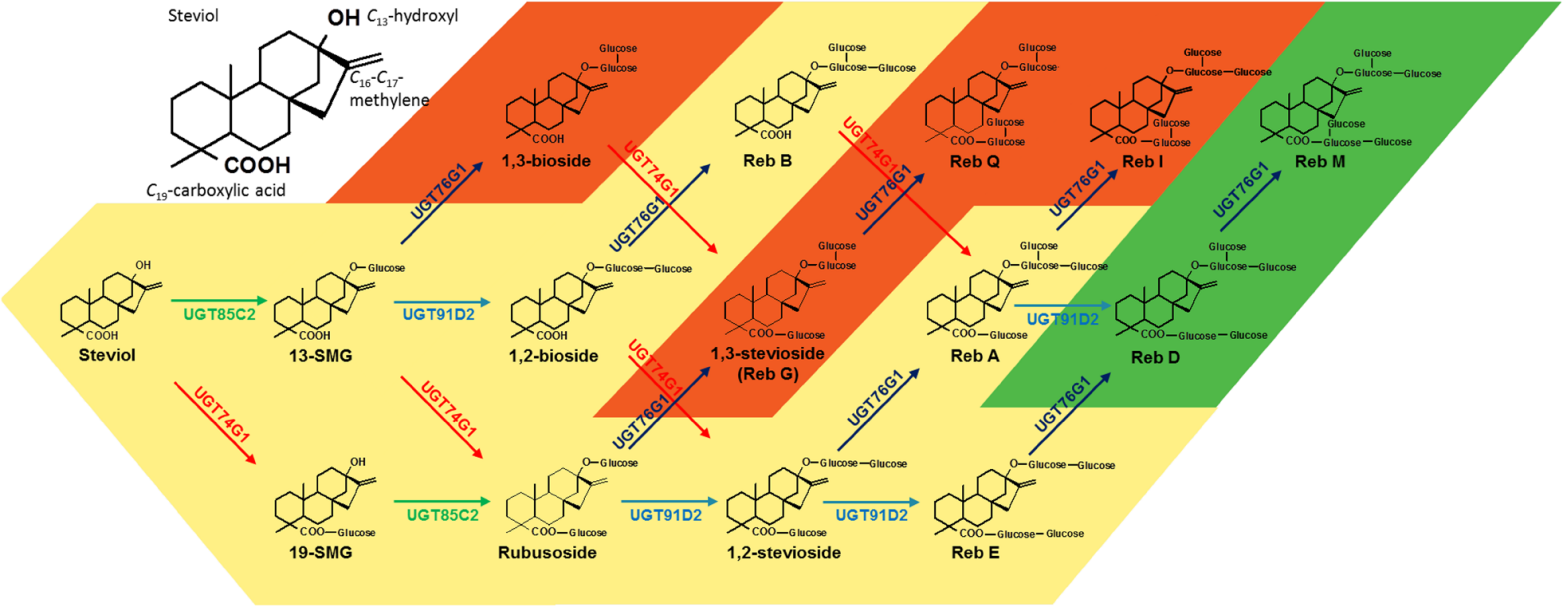
Steviol and the metabolic grid of glucosylation reactions resulting in formation of Reb D and Reb M. *Top left* the structure of steviol with emphasis on its functional groups. Reb D and Reb M are the two desired sweeteners (shown on *green background*). UGT91D2 has not been observed to glucosylate glucoside structures that harbor a glucose residue bound in a 1,3-glucosidic linkage. Formation of the 1,3-bond prior to formation of the 1,2-bond results in production of undesired side-products (shown on *red background*) [13, 15, 16]. UGT76G1 is known to glucosylate Steviol-13-*O*-monoglucoside (13-SMG), rubusoside, 1,2-stevioside and Reb D. In this study, 1,2-bioside, Reb G, Reb A and Reb E were identified as additional UGT76G1 substrates. UGT76G1 catalyzed glucosylation of Reb G and Reb A lead to the formation and structural elucidation of two new steviol glucosides Reb Q and Reb I

Leaves of the “sweet herb” *Stevia rebaudiana* contain a mix of steviol glycosides and have been used as natural sweeteners in South America for centuries. The safety of the steviol glucosides has been ascertained by studies of their catabolism and their use as food additives resulting in their general use in the US as well as in the EU [4–9]. Steviol glucosides consist of a diterpenoid steviol backbone decorated with one to three glucoses at the steviol *C*_13_-hydroxyl and/or *C*_19_-carboxylic acid positions (Fig. 1). The *C*_16_-*C*_17_ methylene double bond present in the natural steviol glycosides is a pharmacophore essential for the sweetness properties of this class of molecules [10]. The maximum sweetness perceived correlates positively to the total number of glucose residues present whereas the substitution of a β-1,2-glucose with an α-1,2-rhamnose, greatly reduces sweetness [11]. The lingering bitter off-flavor of some steviol glycosides is related to their binding to human bitter taste receptors belonging to the class of hTAS2R type receptors whereas sweet taste sensations are mediated by G-protein coupled receptors like heteromers of hTAS1R2 and hTAS1R3 situated in the taste buds [11]. It is the sweet threshold concentration compared to the bitter threshold concentration that determines how steviol glycosides are perceived when consumed as part of a diet.

More than 35 different steviol glycosides have been identified in *S. rebaudiana* [5, 12]. In *S. rebaudiana,* the glucosylation reactions are catalyzed by the action of four UDPG-dependent glucosyltransferases (UGTs): UGT85C2 glucosylates the steviol backbone at the *C*_13_-hydroxyl position forming a β-d-glucoside whereas UGT74G1 glucosylates the *C*_19_-carboxylic acid functional group giving rise to the formation of an ester (Fig. 1) [13]. UGT91D2 and UGT76G1 catalyze glucosylation of the two glucose moieties directly bound to the *C*_13_- and *C*_19_-positions via formation of 1,2-β-d- and 1,3-β-d-glucosidic linkages, respectively. While UGT76G1 is capable of catalyzing 1,3-β-d-glucosylations on both mono- and 1,2-disaccharides attached to the steviol *C*_13_ and *C*_19_-positions, UGT91D2 has not been shown to catalyze 1,2-β-d-glucosylation if a 1,3-disaccharide is already present on the *C*_13_- or *C*_19_-position, potentially yielding a number of 1,3-glucosylated side-products [13–16].

The most abundant steviol glucosides in *S. rebaudiana* are the tri-glucosylated 1,2-stevioside, the tetra-glucosylated rebaudioside A (Reb A) and the tetra-glycosylated rebaudioside C (Reb C) constituting 5–10, 2–4 and 1–2% of the leaf dry weight, respectively [17]. The 1,2-stevioside and Reb A are 250–300 times more sweet than sucrose but possesses a bitter lingering aftertaste [7, 18]. Reb C is only 30 times sweeter than glucose as a result of the attachment of a rhamnose moiety to the glucose moiety at the *C*_13_ position [19]. The less abundant penta-glucoside Rebaudioside D (Reb D) and hexa-glucoside Rebaudioside M (Reb M) and blends thereof have a sweetness potency up to 350 times that of sucrose while having a greatly reduced lingering bitterness [7]. Taste tests at high concentrations registered Reb D among the sweetest and as significantly less bitter than Reb B [20]. Reb M is characterized by a high sweetness intensity, a fast sweetness on-set, a clean taste and with greatly reduced licorice, bitter, sour and astringent aftertaste in comparison to Reb A and other steviol glucosides [7]. These properties render Reb D and Reb M superior targets as high potency natural sweeteners. Reb D and Reb M are only present in the leaves of *S. rebaudiana* in minute quantities (approx. 0.4–0.5% w/w total dry weight) making it impractical and costly to purify these two compounds from the stevia plant for industrial use [7, 21]. As the genes encoding biosynthesis of the steviol glucosides including Reb D and Reb M have been identified, heterologous production of Reb D and Reb M in microorganisms offers an attractive sustainable alternative. With this aim, the steviol glucoside pathway has been successfully expressed in the yeast *S. cerevisiae* [16]. Two advantages of microbial factories are the ability to control the levels of expression of the different pathway genes and the inherent opportunity to use mutational studies to modify the substrate specificity of the enzymes involved.

In the current study, UGT76G1 was mutagenized to improve a *S. cerevisiae* stevia production host for desired combinations of steviol glucosides with a special focus on Reb D and Reb M due to their superior sweetness and organoleptic properties. The challenge faced is that Reb D and Reb M are not formed in a linear pathway from steviol, but are formed as products in a metabolic glycosylation grid and their formation is directly dependent on the catalytic activity of UGT76G1 to convert 1,2-stevioside to Reb A, and Reb D to Reb M. However, UGT76G1 acts like a chameleon enzyme in the pathway, catalyzing different reactions in the metabolic glycosylation grid (Fig. 1). It is known to catalyze the additional conversion of steviol-*C*13-glucoside (13-SMG) and rubusoside into 1,3-bioside and 1,3-stevioside (Rebaudioside G), respectively [16]. Formation of these side-products could diminish the final accumulation of Reb D and Reb M in the production strain (Fig. 1). Increased production of Reb D and Reb M would thus require optimization of UGT76G1 catalytic activity towards glucosylation of 1,2-stevioside, Reb E and Reb D, the substrates for formation of Reb D and Reb M, respectively.

The substrate specificity of UGT76G1 was defined in detail by assaying for activity towards four additional steviol glucosides in vitro. Homology modelling of UGT76G1 was used to predict which residues are likely to contribute to the formation of the binding pocket and the active site. A site-saturation mutation library for each of the selected residues was generated to optimize the geometry of the binding pocket and favor the production of Reb D and Reb M. In this way, 1748 UGT76G1 variants were generated and expressed in *S. cerevisiae.* This resulted in identification of UGT76G1 variants with reduced ability to catalyze the formation of side-products and increased formation of Reb D and Reb M in *S. cerevisiae.*

## Methods

### In vitro characterization of UGT76G1 reactions

Using the lithium acetate transformation protocol [22], p416-GPD [23] and p416-GPD harboring *UGT76G1* were used to transform the protease deficient yeast strain DSY-6 (*MATa leu2 trp1delta63 ura3*-*52 prb1*-*22 pep4*-*3 prc1*-*407*, Dualsystems Biotech, Schlieren, Switzerland). Transformants were selected on plates containing Synthetic Complete media without uracil (SC-ura). Three clones were grown in 1 ml SC-ura media in 96 deep well plates (Ratiolab, Dreieich, Germany) and incubated for 2 days at 30 °C and 400 rpm in an orbital shaker. Aliquots (100 µl) from each culture were subsequently diluted in 1 ml of fresh SC-ura media and incubated for 1 day. To obtain a cell lysate containing UGT76G1, the cultures were centrifuged (3220*g*/20 min) and the pellets lysed with CelLytic™ Y cell lysis reagent (Sigma-Aldrich, St. Louis, Missouri, USA) according to manufacturer’s recommendation. The lysates (6 µl aliquots) were added to a reaction mixture (total volume of 30 µl) consisting of 0.1 M Tris(hydroxymethyl)aminomethane (Tris, pH 8.0), 0.3 mM UDPG (Sigma-Aldrich, St. Louis, MO, USA) and 0.1 mM of either of the following substrates: 1,2-bioside; rubusoside; 1,2-stevioside; rebaudioside E; rebaudioside A and rebaudioside D (LGC Standards, Wesel, Germany). Samples for time-course experiments were taken after 0, 1, 2 and 18 h. Reactions were stopped by adding 25 µl dimethyl sulfoxide (DMSO) to a 25 µl aliquot of the reaction mixture.

### Steviol glycoside analysis by LC–MS

Liquid chromatography mass spectrometry (LC–MS) analyses were performed using an Ultimate 3000-RS UPLC (Dionex Thermo Fisher Scientific) equipped with a Waters Acquity UPLC ®BEH C18 column (2.1 × 50 mm, 1.7 µm particles, 130 Å pore size, flow rate: 0.4 ml/min, column temperature of 35 °C) coupled to a Quantum Access TSQ triple quadrupole mass spectrometer (Thermo Fisher Scientific) with electrospray ionization (ESI) operated in positive mode. The mobile phases were: A, H_2_O with 0.1% formic acid (HCOOH) and B, acetonitrile (CH_3_CN) with 0.1% HCOOH. The gradient used was: 0.0–4.0 min linear gradient 25–47% B; 4.0–5.0 min, linear increase 47–100% B; 5.0–5.5 min, 100% B; re-equilibrate to the gradient starting ratio). The masses corresponding to Steviol-*C*_13_- and -*C*_19_-glucosides (13- and 19-SMG;* m*/*z* 481.3 [M+H]^+^ and 503.3 [M+Na]^+^), Steviol + 2 glucoses (Rubusoside, 1,2- and 1,3-bioside; * m*/*z* 665.3 [M+Na]^+^), Steviol + 3 glucoses (1,2-stevioside, Reb B and G; * m*/*z* 827.4 [M+Na]^+^), Steviol + 4 glucoses (Reb A, E and Q; * m*/*z* 989.4 [M+Na]^+^), Steviol + 5 glucoses (Reb D and I; * m*/*z* 1151.5 [M+Na]^+^) and Steviol + 6 glucoses (Reb M; * m*/*z* 1313.5 [M+Na]^+^) were monitored using single ion monitoring (SIM) and quantified by comparison with authentic standards, when available.

### Homology modeling of UGT76G1 and docking of steviol glucosides

Homology modelling of UGT76G1 was performed using the ORCHESTRA module in Sybyl-X 2.0 (Certara, St. Louis MO) using the following PDB-files as templates: 2PQ6, 2C1X, 3HBF and 2VCE. The ligands in PDB:2VCE were used during the generation of the main- and side-chains and removed prior to energy minimization with an AMBER FF99 force field. Model geometry and quality were checked with the web servers molprobity [24, 25] and ProQ [26]. The uridine diphosphate glucose (UDPG) fluorinated-sugar donor analog UDP-2-deoxo-2-fluoro glucose (UDP-2FGlc) from PDB: 2VCE was imported into the UDPG binding site of UGT76G1 prior to the steviol glucoside acceptor substrates. Models of Reb D and Reb M were prepared using the SybylX small molecule builder and docked into the active site of the enzyme with the Surflex-Dock GeomX module allowing for protein flexibility. The sites targeted for site-saturation mutagenesis were determined by selecting residues within a 5 Å distance of Reb D and Reb M in the docking analysis with exclusion of residues that were found to be 100% conserved in the PDB-templates.

### Design of the *UGT76G1* site-saturation library vectors

For each of the selected amino acids in the active site to be mutagenized, a site-saturation library was generated by BaseClear (Leiden, The Netherlands). A total of 38 *UGT76G1* libraries were generated by PCR using NNS-degenerate primers, with N designating that A, C, G, or T nucleotides are present in the first two codon positions, and S designating the presence of G or C nucleotides in the third codon position for the position to be site-saturated. The mutagenized constructs were then cloned into p416-GPD using the restriction sites SpeI and XhoI. The quality of the libraries was assessed by sequencing 96 colonies for each of two libraries to verify the presence of >17 variants in the library pool. In addition, 2–3 colonies of each of the remaining 36 libraries were sequenced to ensure the presence of variants at the desired positions.

### Determination of the in vivo activity of UGT76G1 site-saturation library in steviol glucoside producing *S. cerevisiae* strain

Using the lithium acetate protocol [22], the 38 *UGT76G1* site-saturation libraries were transformed into a *S. cerevisiae* strain harboring all the genes required for Reb M biosynthesis except *UGT76G1* [16]. Forty-six transformants and two wild-type UGT76G1 controls for each library site were inoculated in 1 ml SC-ura and grown for 4 d (400 rpm orbital shaking, 30 °C). Aliquots (50 µl) of cells from each culture were added to 50 µl DMSO, heated at 80 °C for 10 min, and centrifuged (3220*g*/10 min) to obtain a clear supernatant. LC–MS was used to analyze the extracts for their content of Reb D and Reb M as described above. The Genejet Plasmid Miniprep Kit (Thermo Fisher Scientific, Waltham, Massachusetts, USA) was used to purify plasmids from yeast colonies expressing UGT76G1 variants of interest. The procedure was performed according to manufacturer’s recommendations except for the introduction of a bead-bashing step to break the cells after the addition of the resuspension buffer. Glass beads [one-third (v/v), 425–600 µm, Sigma] were added to the resuspended cultures which were then treated for 20 s at max speed in a Thermo Electron FastPrep FP120 Cell Disruptor (Thermo Fisher Scientific, Waltham, Massachusetts, USA). Selected UGT76G1 variants were sequenced by Macrogen Europe, (Amsterdam, The Netherlands) and the plasmids were re-transformed into the *S. cerevisiae* producing strain lacking UGT76G1 and incubated as described above in triplicate to eliminate the possibility of phenotypes observed due to genetic background changes. Accumulation of the steviol glucosides presented in Fig. 1 was monitored by LC–MS as described above.

## Results

### In vitro characterization of UGT76G1’s activity on steviol glucosides

**Fig. 2:**
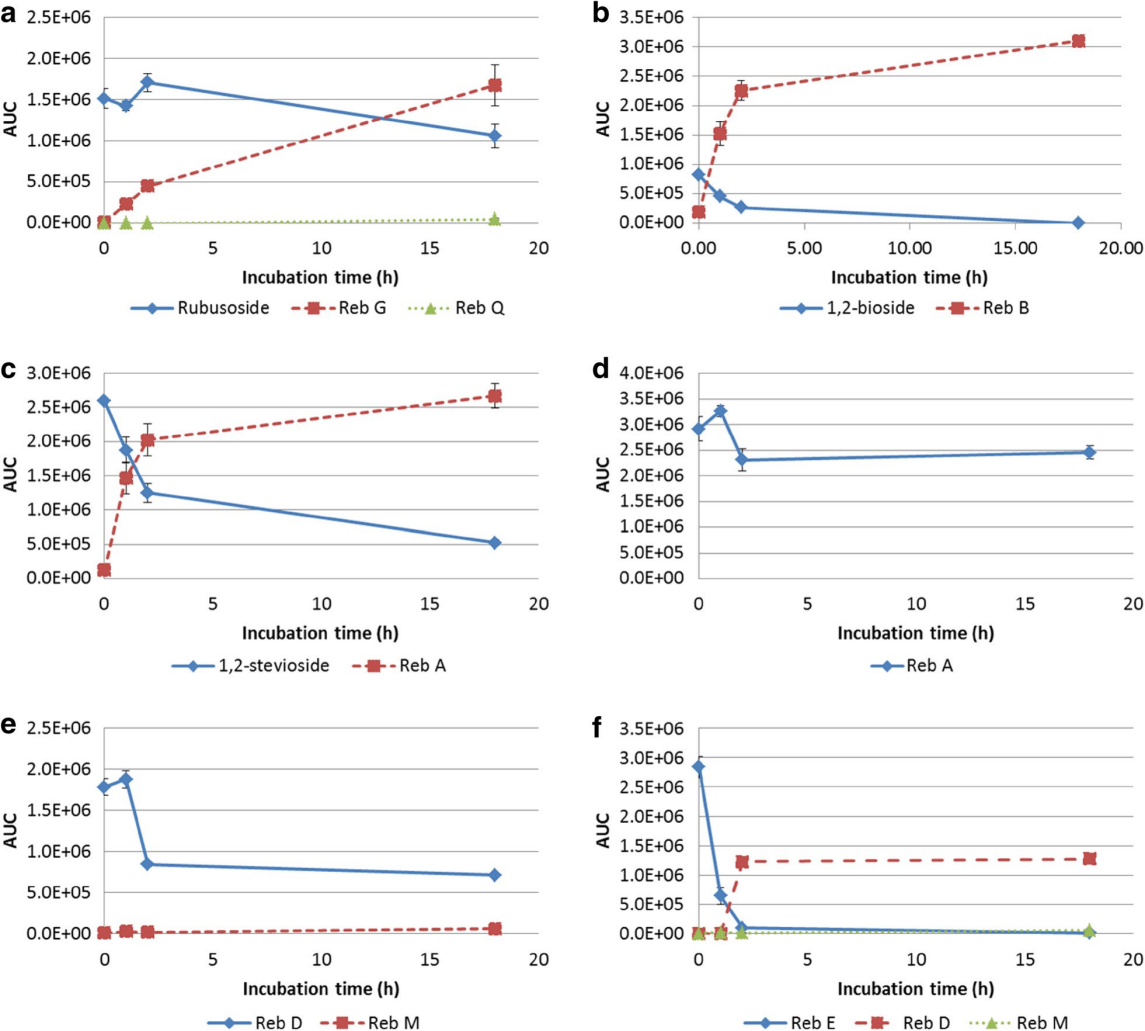
1.3-Glucosylation reactions catalyzed by wild-type UGT76G1. Substrates tested were: **a** rubusoside, **b** 1,2-bioside, **c** 1,2-stevioside, **d** Reb A, **e** Reb D and **f** Reb E. Each substrate was administered at an initial concentration of 0.1 mM. Substrate conversion and formation of 1,3-mono-, di- and tri-glucosylated products are shown in *blue*, *red* and *green* traces, respectively. Reb I resulting from glucosylation of Reb A is not depicted due to partial co-elution with Reb A and 1,2-stevioside which prevents integration of the Reb I peak in the LC-MS results. The AUC units depicted do not offer the ability to make quantitative comparisons between different components

To identify all potential UGT76G1 substrates, the substrate specificity of wild-type UGT76G1 obtained as a DSY-6 lysate was tested in in vitro assays in the presence of steviol glucosides and UDPG. The time course experiments (Fig. 2) demonstrated that, besides glucosylating 13-SMG (not tested in this study), 1,2-bioside 1,2-stevioside and Reb D, the enzyme was capable of glucosylating rubusoside, Reb E, Reb G and Reb A, catalyzing 1,3-glucosylations at the *C*_13_- as well as *C*_19_-positioned glucose moieties on the steviol backbone (Fig. 1).

Based on percentage changes in area under the curve (AUC) values, the results demonstrate that UGT76G1 was much more efficient at catalyzing 1,3-glucosylations of the glucose moiety linked directly to the *C*_13_-position of the steviol ring system in comparison to glucose moieties located at the *C*_19_-position. This was manifested in the almost complete glucosylation of 1,2-bioside, 1,2-stevioside and Reb E within an 18 h period with the best substrate for glucosylation at the *C*_19_ position being Reb D (Fig. 2).

### Identification of novel steviol glucoside side-products

LC–MS analysis on reaction mixtures containing cell lysate from *S. cerevisiae* expressing wild-type UGT76G1 and added rubusoside or Reb G revealed the production of a novel compound with a mass corresponding to a steviol tetra-glucoside, while incubation with 1,2-stevioside and Reb A gave rise to a compound corresponding to a novel steviol penta-glucoside. Since UGT76G1 has only been shown to catalyze 1,3-glucosylations, the tetra-glucoside is expected to be a steviol glucoside with an attached 1,3-glucose moiety positioned at both the *C*_13_- and *C*_19_-glucoses. We named this novel compound rebaudioside Q (Reb Q, Fig. 1). In a similar manner, the penta-glucoside is expected to be rebaudioside I (Reb I, Fig. 1, [12, 27]). In our experimental setup, Reb I partly co-elute with 1,2-stevioside and Reb A preventing determination of a specific AUC for this compound (Additional file 1: Figure S1).

### In silico analysis

**Fig. 3:**
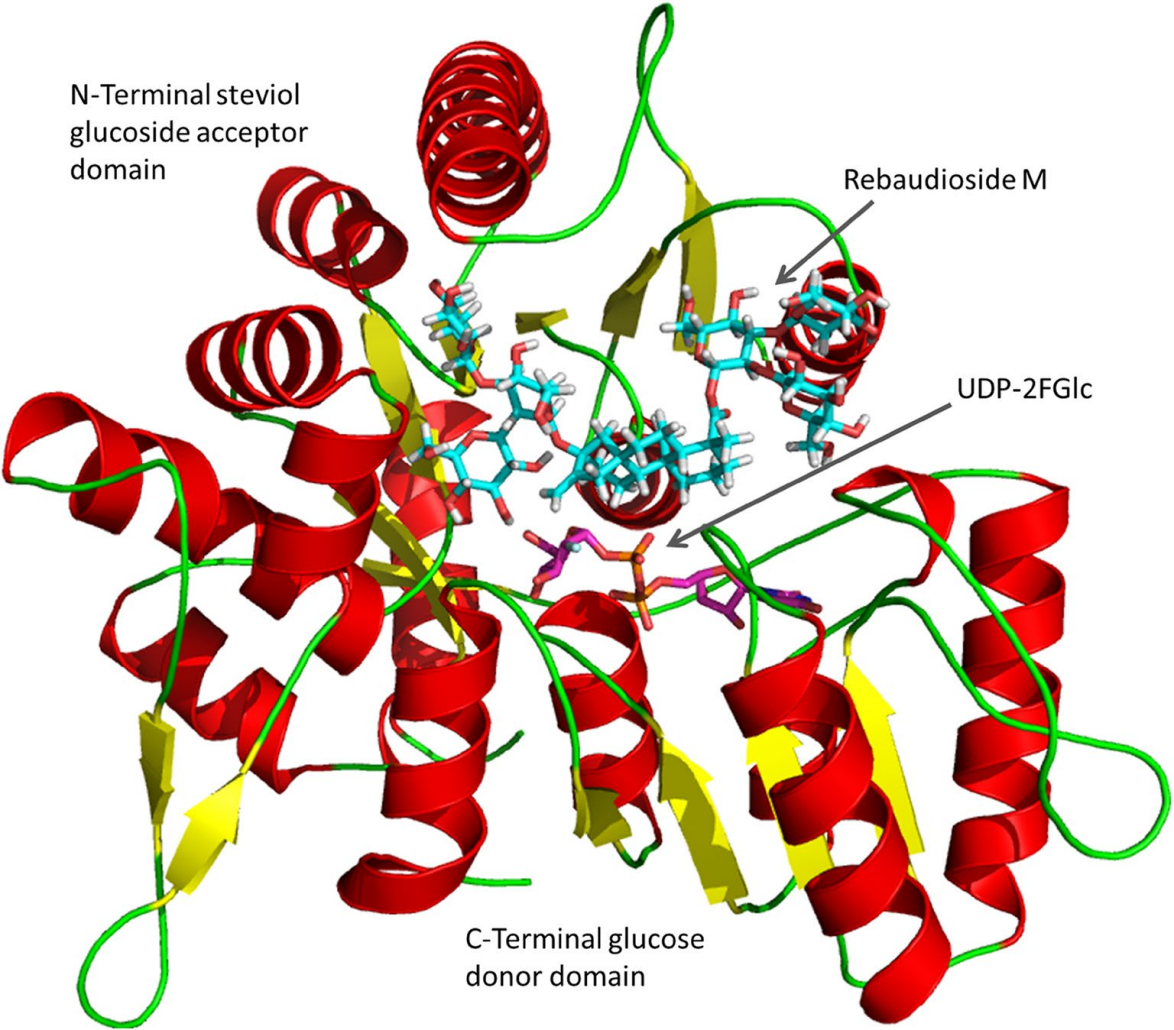
UGT76G1 homology model with Reb M (*cyan*) and the UDPG fluoride analog UDP-2FGlc (*magenta*) docked in the active site. The model shows the parallel β-sheets (*yellow arrows*) and the α-helices (*red*) of the two Rossmann folds making up the N- and C-terminal domains characteristic of family 1 UGTs

Homology modelling of UGT76G1 was performed based on the crystal structures for the plant UGTs with the highest sequence identity (ID). These were MtUGT85H2 (PDB: 2PQ6, ID = 30.5%), VvGT1 (PDB: 2C1X, ID = 29.4%), MtUGT78G1 (PDB: 3HBF; ID = 27.0%) and AtUGT72B1 (PBD: 2VCE, ID = 27.3%) [28–31] (Fig. 3).

UGT76G1 folded into the expected two Rossmann fold domain structure characteristic of GT-B folded Leloir (nucleotide-dependent) glycosyl transferases [32–34] with Ile15-Glu236 constituting the N-terminal aglycon acceptor domain and Ser255-Val456 constituting the C-terminal UDPG-donor domain connected by the linker Ile237-Ser254. The quality of the UGT76G1 homology modelling was evaluated by calculating Molprobity score, LGscore and MaxSub values with the webservers Molprobity and ProQ [24–26]. The Molprobity score was 1.89, LG score 5.761 and the MaxSub value 0.201. These values all indicate a high probability of correct folding and thus high quality of the obtained UGT76G1 model.

**Fig. 4:**
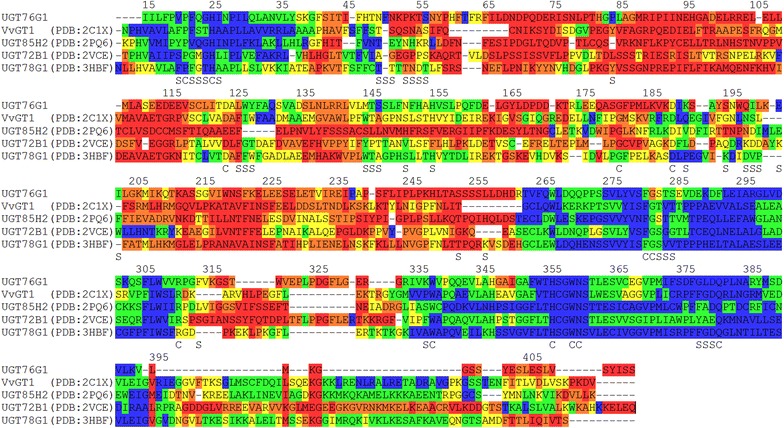
RMSD between the tertiary structure of UGT76G1 and the four crystal based structural templates. *Blue* <1 Å; *Green* 1–2 Å; *Yellow* 2–3 Å; *Orange* 3–4 Å and *Red* >4 Å. Numbering is according to the UGT76G1 amino acid sequence. *S* UGT76G1 residues selected for site-saturation mutagenesis. *C* residues considered for site-saturation mutagenesis but found to be fully conserved

Superimposition of UGT76G1 with the four crystal based templates showed that the tertiary structure of the C-terminal domain was highly conserved between UGT76G1 and the templates, reflecting its major function as the UDPG binding domain. The N-terminal domain, responsible for binding the glucose-acceptor substrates, was more variable with several regions that deviated more than 4 Å between their respective Cα-atoms. These differences were described to the vast structural differences in sugar-acceptor substrates (Fig. 4).

The high structural identity between the C-terminal domain of UGT76G1 and the four crystal based structures and the known conformation of the sugar donor in the crystal based structures enabled docking of the catalytically inactive fluorinated UDPG-analog UDP-2FGlc from the crystal structure of UGT72B1 (PDB: 2VCE; Shao et al. [35]) into UGT76G1. This constrained the available space in the binding pocket before introduction of the steviol glucosides. Forty-nine amino acid residues in the binding pocket were considered relevant for Reb D and Reb M binding and activity. Eleven of these residues were omitted from the site-saturation library screen because these amino acids were found to be fully conserved within UGT76G1 and all four templates used and therefore expected to be important for catalytic function. These conserved residues include His25 and Asp124, the two amino acids presumed to be responsible for transferring the sugar moiety from the donor UDPG molecule to the acceptor substrate [28–31] and Trp338-Gln381 which are part of the highly conserved plant secondary product glycosyltransferase (PSPG)-motif responsible for UDPG binding [36]. Finally, Pro21 is expected to stabilize a loop prior to the α-helix containing the catalytic His25, while Phe281, Gly282 and Arg311 form part of the hydrophobic core in the C-terminal domain. The remaining 38 amino acid sites were targeted for site-saturation mutagenesis (Fig. 4).

### In vivo site-saturation mutagenesis screen

The 38 residues selected from the in silico analysis were individually mutagenized with NNS-primers and transformed into the *S. cerevisiae* strain harboring all genes necessary for Reb M production except UGT76G1. Forty-six independent transformants were screened for each of the 38 selected sites along with two wild-type controls for each site, resulting in a total of 1824 clones. Using NNS-degenerate primers, a theoretical codon coverage of 85% was achieved resulting in an average of 17 different amino acids being represented per position [37]. Reb D and Reb M production was measured in *S. cerevisiae* cultures from all the clones expressing the UGT76G1 variants. Within the 76 *S. cerevisiae* clones expressing wild-type UGT76G1, the accumulation of Reb D and Reb M showed variation in the accumulation level of Reb D between 0.18 and 12.17 µM and variation in the Reb M levels between 0.4 and 43 µM, whereas the ratio between the two rebaudiosides was constant with a Reb D/Reb M ratio of 0.25 ± 0.02 (Additional file 2: Figure S2).

**Fig. 5:**
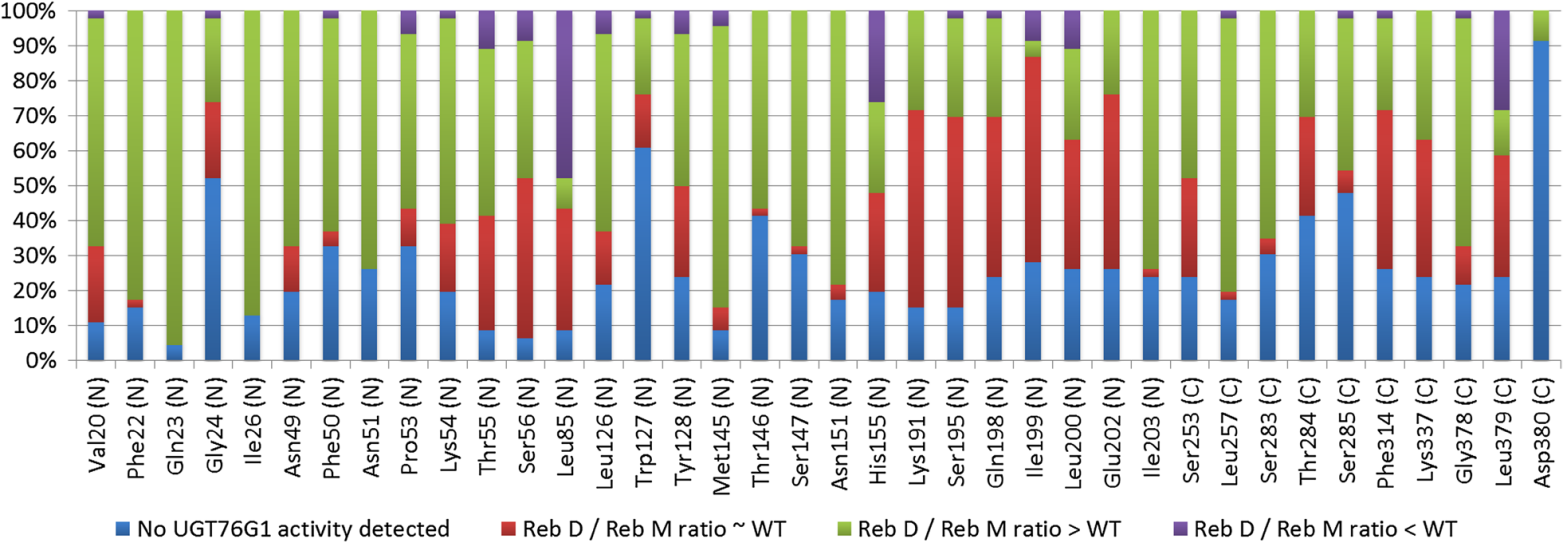
Effect of site-saturating selected amino acid residues the UGT76G1 binding pocket on activity and Reb D and Reb M accumulation. 46 colonies containing UGT76G1 variants were screened for each of the selected 38 different amino acid positions compared to colonies expressing the wild-type enzyme. The N and C in parenthesis next to a residue denotes whether the residue resides in the N- or C-terminal domain of UGT76G1

Analysis of the 38 residues that were site-saturated (Fig. 5) showed that mutagenesis of 19 of the targeted residues exhibited a tendency for generating UGT76G1 variants that increased the average Reb D/Reb M ratio as evaluated from the 46 clones screened for each site. In contrast, analysis of the clones from the target sites Leu85, His155 and Leu379 predominantly gave rise to UGT76G1 variants with a decreased Reb D/Reb M ratio. The ratio between the two rebaudiosides produced was characterized as being different from the wild-type ratio if it was more than two standard deviations above or below.

**Fig. 6:**
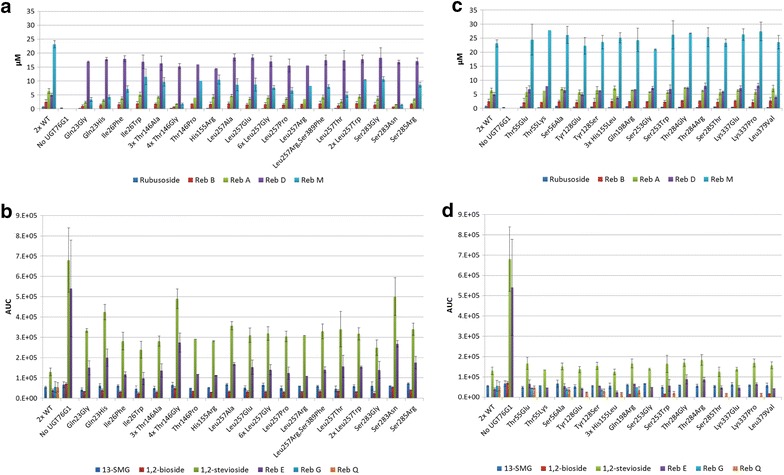
LC-MS analysis of the steviol glucosides present in the *S. cerevisiae* clones expressing UGT76G1 variants. **a**, **b** UGT76G1 variants found to increase Reb D accumulation (High Reb D variants); **c**, **d** UGT76G1 variants examined for increased Reb M accumulation (High Reb M variants). The number of clones identified to harbour the same UGT76G1 mutation is shown. For the three variants Thr146Pro, Leu257Arg and Thr55Lys, no *error bars* are shown because only a single sample of each was screened due to background strain variation during transformation. Two sets of wild-type controls (WT) were analysed in triplicate with each test. Steviol, steviol-19-*O*-glucoside (19-SMG) and 1,3-bioside were not detected in any of the samples. The steviol glucosides in **a** and **c** were quantified based on authentic reference compounds whereas those in **b** and **d** were assessed by their respective AUC units (area under curve)

The 47 *S. cerevisiae* clones that produced more than 23 µM Reb D and the 47 clones that produced more than 34 µM Reb M (Additional file 2: Figure S2) were re-tested in triplicate. Of these, the 30 clones that showed the highest accumulation of Reb D (Fig. 6a, b) and the 18 clones that showed highest accumulation of Reb M (Fig. 6c, d) were sequenced and selected for further study. These clones were defined as High Reb D mutants and High Reb M mutants, respectively. Rubusoside, Reb B, A, D and M were quantified based on authentic standards, whereas the amounts of 13- and 19-SMG, 1,2- and 1,3-bioside, 1,2-stevioside and Reb G, E and Q present were assessed from the LC–MS profiles and the AUC units recorded for each of the compounds.

A total of thirty-three unique UGT76G1 variants were identified among the 30 high Reb D variants and 18 high Reb M variants investigated. Unexpectedly, a Ser389Phe mutation, which was not at a residue selected for site-saturation mutagenesis appeared together with a Leu257Arg substitution in a high Reb D variant.

Analysis of the *S. cerevisiae* strains selected for increased Reb D accumulation showed up to four times the content of Reb D by increasing the concentration from 5 µM in the strain expressing wild-type UGT76G1 to above 18 µM in several of the strains containing site-saturated UGT76G1 variants (Fig. 6a). In the same strains, the accumulation of Reb M was reduced from 23 µM in the strain expressing wild-type UGT76G1 to 2 µM in strains expressing UGT76G1_Thr146Gly_ and UGT76G1_Ser283Asn_. Accumulation of 1,2-stevioside and Reb E increased from AUC-levels 130 × 10^3^ to 681 × 10^3^ and 49 × 10^3^ to 541 × 10^3^, respectively, between strains expressing wild-type UGT76G1 and a strain that does not express UGT76G1. The 1,2-stevioside and Reb E are both known substrates for UGT76G1 and the high Reb D mutants showed elevated levels of these two steviosides. Other UGT76G1 substrates such as 13-SMG, rubusoside and Reb A did not accumulate in increased concentrations in any of the mutants tested. Likewise, the unwanted side-products 1,3-bioside, Reb G and Reb Q did not increase (Fig. 6b, d).

Analysis of the *S. cerevisiae* clones selected for increased Reb M accumulation identified five UGT76G1 mutations (Thr55Lys, His155Leu, Thr284Gly, Lys337Glu and Lys337Pro) that offered increased production of Reb M. The increase in production was significant but not nearly as remarkable as observed for Reb D. The mutant clones all showed similar levels of 13-SMG, Reb A, Reb B and rubusoside accumulation compared to clones expressing wild-type UGT76G1. 19-SMG and 1,3-bioside were not detected in any of the samples while a general increase in Reb D level was observed in the strains expressing high Reb M UGT76G1 variants (Fig. 6c, d).

### Effect of site-saturating the UGT76G1 binding pocket, as predicted by homology modelling

In this study, a high quality homology model of UGT76G1 was built based on four solved UGT crystal structures and assessed to show a high likelihood of correct folding. Docking of Reb D and Reb M into the active site of UGT76G1 identified 38 non-conserved amino acid residues within a distance of less than 5 Å from the bound steviol glucosides. Of these, 28 residues were situated within the N-terminal part of UGT76G1 while only 10 resided in the C-terminal domain. This is in agreement with earlier observations indicating that the N-terminal domain is primarily responsible for sugar-acceptor interactions whereas the PSPG- motif in the C-terminal binds the activated sugar-donor [38–40].

**Fig. 7:**
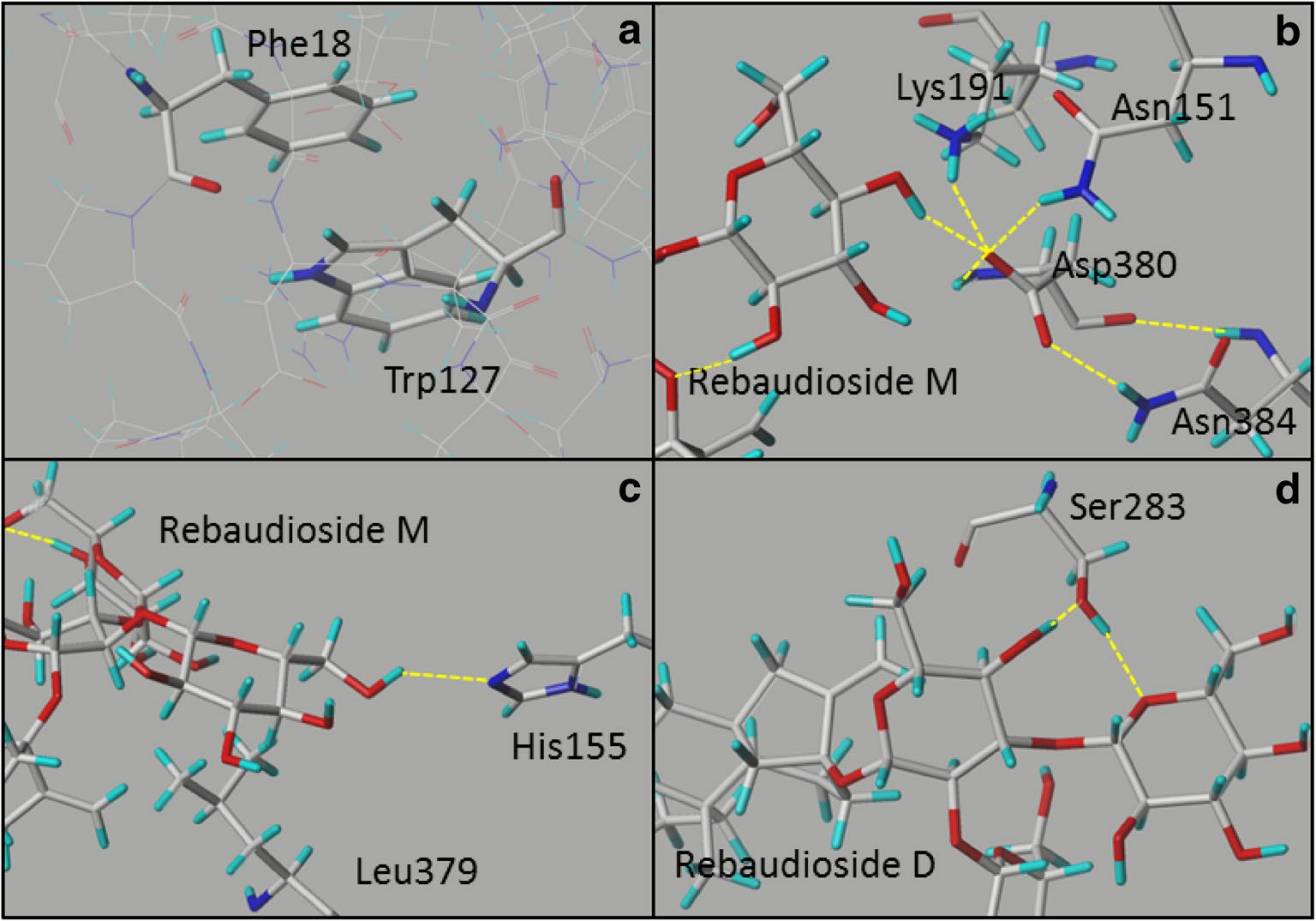
Residue interactions as predicted by UGT76G1 homology modelling. **a** π-stacking between Phe18 and Trp127 **b** Hydrogen bond formation from Asp380 to Asn151, Lys191, Asn384 and to a Reb M glucose moiety. **c** Hydrogen bond formation from His155 with Reb M and the Leu379 directing planar conformation of the glucose residues of the stevioside. **d** Hydrogen formation from Ser283 to the *C*_13_-glucose moieties

Out of the 38 residues that were subjected to site-saturation mutagenesis, Gly24, Trp127 and Asp380 showed the highest sensitivity to mutagenesis with 52, 61 and 91% of the tested yeast clones showing no UGT76G1 activity, respectively, indicating that these residues are important for the catalytic function (Fig. 5). Of these three, Gly24 is positioned next to the catalytic His25 and the substitution of the small side-chain of glycine with a bulky or positive charged side-chain could interfere with the stabilizing interaction of Asp124 and His25. The importance of the Gly24 residue was also apparent from the crystal structures used as templates for the homology model, where only glycine or threonines were found at this position. Trp127 forms a π-stack with Phe18 (Fig. 7a) stabilizing the N-terminal domain of UGT76G1, while Asp380 is part of the glycosyltransferase PSPG-motif. Asp380 was included in the screen because it was not 100% conserved with VvGT1, the exception having a glutamate at this position. That 91% of the Asp380 mutated clones had no activity (Fig. 5) indicates that a negative charge at this position is critical for activity. In the homology model of UGT76G1, the carboxylic acid of Asp380 interacts by hydrogen bonding to the side-chains of Asn151, Lys191, Asn384 and to a glucose residue of Reb M. Asp380, Asn151, and Lys191 form a triad that stabilizes that region of the UGT (Fig. 7b). It is therefore not surprising that mutating Asp380 disrupted the stability of UGT76G1 and gave rise to a high number of clones with no activity. Although part of the triad, the residues between Lys191 and Glu202 were less susceptible to loss of enzyme function as more than 50% of the clones displayed wild-type ratios of Reb D and Reb M accumulation (Fig. 5).

In the primary screen, it was found that the SSL’s for the three positions: Leu85, His155, and Leu379 resulted in several UGT76G1 colonies with increased Reb M/Reb D ratio. None of the Leu85 colonies was selected for further study due to insufficient accumulation of Reb M, while three colonies from the His155 and one colony from the Leu379 SSL were selected for increased Reb M/Reb D ratio. His155 forms a hydrogen bond to the 1,2-glucose attached to the *C*_13_-position of the steviol backbone while the di-methyl group on Leu379 maintains the three glucose moieties in a planar conformation and interacts with the steviol *C*_16_-methylene group (Fig. 7c). In those UGT76G1 variants where increased production of Reb M was observed, these interactions would appear to have been favoring Reb D to Reb M conversion.

Yeast expressing UGT76G1 variants with the mutations Lys337Pro, and the single successful replicate of Thr55Lys, showed an increase in Reb M accumulation of approximately 20% when compared to the wild-type (Fig. 6c). The side-products generated by glucosylation of rubusoside to Reb G and Reb Q in these mutants were almost abolished with no Reb G detected and one-third of the Reb Q levels present in strains expressing UGT76G1_Lys337Pro_ compared to wild-type UGT76G1. Thr55 and Lys337 are positioned at the entrance of the binding site and could regulate the binding and the dissociation of the sugar-acceptor ligands. Furthermore the hydroxyl side chain of Thr55 forms a hydrogen-bond with the 1,3-glucose bound to the *C*_19_ attached glucose on the steviol backbone. The Thr55Lys substitution eliminates formation of a hydrogen-bond to Reb M. The hydrocarbon chain of Lys337 interacts with the hydrophobic sidechain of Leu257. Changing Lys337 into a proline could help stabilize the hydrophobic interaction. UGT76G1_Thr284Gly_ gave increased accumulation of all steviol glucosides except rubusoside and the side-products Reb G and Reb Q.

High Reb D mutants were found to primarily arise as a result of the reduced ability of UGT76G1 to catalyze the Reb D to Reb M reaction. Other reactions were also inhibited as evident by increased accumulation of the UGT76G1 substrates 1,2-stevioside and Reb E, with 1,2-stevioside AUC increasing up to four times that of the clones expressing wild-type UGT76G1, while Reb E AUC increased up to seven times (Fig. 6b). The additional accumulation of Reb D observed in the high Reb M mutants may reflect inhibition of the sequential UGT76G1 catalyzed side-reactions from rubusoside to Reb G and Reb Q, and the reduced Reb B accumulation in the cells (Fig. 1). The UGT76G1 variants Ile26Trp and Ser283Gly showed the highest Reb D/1,2-stevioside ratio, indicating that these mutations have low activity on Reb D without mitigating the 1,2-stevioside to Reb A reaction. Docking Reb D into the UGT76G1’s binding pocket showed that the hydroxyl group of Ser283 interacts in hydrogen bond formation with the *C*_13_-glucose moiety as well as to the ring-oxygen of the 1,3-bound glucose (Fig. 7d). The Ser283Gly mutation removed these hydrogen-bonds, which could reduce the binding affinity of Reb D. The 1,2-stevioside does not possess a 1,3-bound glucose moiety attached to the 13-*O*-glucose and the reduction in binding affinity would not be as pronounced with this substrate. Ile26 is part of the hydrophobic core of the N-terminal domain, but the side-chain is not directly interacting with the ligands indicating that the effect of the mutation is due to changes in structural flexibility or stability.

The UGT76G1 variant Leu257Gly was identified six times and gave rise to almost four times as high accumulation of Reb D compared to wild-type UGT76G1. The UGT76G1 variants resulting in the highest Reb D/Reb M ratio were Thr146Gly and Ser283Asn. These variants had a Reb D/Reb M ratio that was up to 50 times higher compared to the wild-type UGT76G1, from a Reb D/Reb M ratio of 0.25 in the wild-type to almost 12 in UGT76G1_Ser283Asn_. Thr146 is engaged in hydrogen-bond formation to the catalytic His25 while Leu257 is part of a highly flexible outer loop that only interacts with Reb M. The Ser283Asn variant reduces the conversion of Reb D to Reb M. The decreased activity could be due to reduction in the binding pocket size, restricting access to Reb D.

The Ser389Phe mutation identified together with Leu257Arg gave a higher Reb D accumulation than Leu257Arg alone, despite its position on the enzyme surface away from the ligand binding site and Leu257. These results open up for generation of double mutants to investigate their effect on the Reb D/Reb M ratio, contra the general catalytic activity of UGT76G1. Despite the increase in Reb D observed for the Reb D variants, a large pool of precursor glucosides was also observed with these variants. This pool of precursors could potentially be better utilized if the Reb D mutations were combined with one of the catalytically more active Reb M mutations, further increasing the accumulation of Reb D.

## Discussion

The in vitro investigation of wildtype UGT76G1 demonstrated that this enzyme has a broad substrate specificity catalyzing glucosylation of numerous steviol glucosides in addition to Reb D and Reb M (Fig. 1). This was expected since UGTs are known to be promiscuous, catalyzing glucosylation reaction on classes of substrates, rather than single molecules [35, 41]. In this case, these additional reactions can give rise to the side-products 1,3-bioside, 1,3-stevioside (Reb G), Reb Q and Reb I, none of which could be converted to Reb D or Reb M by the tested UGT. Hence engineering the substrate specificity of UGT76G1 is imperative for obtaining variants that can be used to improve Reb D and Reb M production.

The key to engineering the substrate specificity of enzymes is the ability to identify the residues involved in substrate selectivity from the vast array of amino acids substitution and their possible combinations within a single protein. Residue identification has traditionally been done through the analysis of crystal structures as e.g. done by He et al. [42], who found that it was possible to change the regio-selectivity of UGT71G1 on quercetin and obtain activity towards genistein by site directed mutagenesis of key residues, such as UGT71G1_Phe148Val_ and UGT71G1_Tyr202Ala_ in the binding pocket [42].

However, when crystal structures are not available, previous studies had to rely on random mutagenesis in combination with effective high-throughput screens or selection schemes. Nonetheless, in some cases neither a crystal structure of the target protein is available, nor is it possible to identify a selection pressure for a high-throughput screening method. To remedy this and allow for optimization of such proteins the search space would have to be reduced so the number of samples to be analyzed can more readily be handled. A way to achieve this has been through the advent of homology modelling.

Homology modelling of UGT76G1 was based on plant UGT crystal structures [28–31] which enabled predictions of the residues located in the binding pocket of the UGT.

When the UGT71G1 crystal structure and UGT76G1 homology model is superimposed, Phe148 and Tyr202 from UGT71G1 are found to be structurally close to Asn151, His155 and Ile203 UGT76G1 SSL’s. Although no colonies were selected for further studies for the Asn151 and Ile203 SSL, these positions were found to yield 70–80% colonies with a change in specificity towards Reb D, while the UGT76G1_His155_ SSL changed specificity towards Reb M (Fig. 5) indicating the importance of these positions in UGT’s for substrate selectivity. Despite the high degree of secondary structural homology between UGTs [34], it is not currently possible to reliably predict the exact orientation and interactions of the UGT76G1 residue side-chains with the ligands due to (1): the diversity and flexibility of the N-terminal sugar-acceptor domains in UGTs and (2): that there are currently no crystal structures of UGTs with steviol glucosides co-crystallized in the binding pocket, this problem was observed with UGT94B1 [39], where homology modelling made it possible to change the sugar-donor specificity of UGT94B1 from UDP-glucuronic acid to UDPG by site-directed mutagenesis, but was unable to change the sugar acceptor specificity from cyanidin 3-*O*-glucoside to delphinidin 3-*O*-glucoside by point mutations in the acceptor binding pocket, despite changing residues seen to be involved in specificity such as Asn123 corresponding to the Leu126 SSL in UGT76G1 and Ile187 corresponding to Tyr202 in UGT71G1 and the Ile203 SSL in UGT76G1 as described above. Therefore changing UGT76G1 sugar acceptor substrate specificity to improve Reb D and Reb M accumulation while simultaneously reducing side-product reactions necessitated a directed evolution approach. Early attempts at altering the glycosyltransferase substrate specificity relied on error prone PCR (epPCR) due to the lack of structural knowledge of the enzyme at the given time, and high throughput screening using an fluorescent aglycone to enable directed evolution of the sugar donor specificity [43]. The residues identified in this screen was then used to continue the directed evolution towards a non-fluorescent aglycone by performing site-saturation mutagenesis on three residues relying on LC–MS for analysis [44]. Here site-saturation mutagenesis was selected for the directed evolution of UGT76G1, since it decreases sample space by function of its controlled ratio of nucleotides at each given position of a codon, giving a better coverage of the mutation space for each amino acid investigated. Furthermore, site-saturation mutagenesis can be targeted to specific regions of the enzyme such as the binding pocket to further reduce sample space as enabled by homology modelling.

Further reduction of the library size was achieved using multiple sequence alignment to omit 11 residues found to be fully conserved, such as the catalytic His-Asp dyad and a number of residues part of the PSPG-motif, as these were assumed to primarily yield loss of function variants. This was also what was found by He et al. [42], who got inactive variants when mutating Trp339, His357, Trp360, Asn361 and Gln382 in the UGT71G1 PSPG motif, corresponding to residues that was omitted from the UGT76G1 screen due to full conservation of the residues [42] The importance of these residues is also seen in this study, by the loss of activity in 91% of the UGT76G1variants located at Asp380 which is part of the PSPG-motif.

By screening 46 colonies for each of the 38 site-saturation libraries, it was possible to gain an 85 and 98% chance of identifying the most, or one of top two, most beneficial mutations respectively, for each of the 38 residues mutagenized in the binding pocket ensuring optimal geometry and amino acid properties for Reb D and Reb M production [37]. Without homology modelling support, attaining the same coverage for each of the 456 residues of UGT76G1, would have required screening 20,976 variants. In the absence of a crystal structure, homology modelling assisted site-saturation mutagenesis of UGT is the most thorough method for modulating substrate specificities currently available, whilst simultaneously ensuring a minimum amount of screening redundancy.

While general purpose, high throughput screening methods are becoming available for glycosyltransferases, none of the current methods were applicable for this study, either because the established screening methods rely on fluorescent substrate analogs which are not suitable for substrate specificity screens [45–47], pH changes which would be indistinguishable from the yeast endogenous metabolism [48], or detection of uridine diphosphate (UDP) produced as a function of UGT activity which would cause background noise due to the UDP found in yeast cells [49–52]. Furthermore, none of these assays would be able to differentiate between the two sequential reactions UGT76G1 is capable of performing on rubusoside, stevioside and reb E. Beside this, investigating 1748 variants for eight different reactions with an in vitro screen would be significantly more laborious than LC–MS analysis. Instead of investigating the variants on individual substrates, performing the UGT76G1 directed evolution in vivo using a yeast rebaudioside-production strain as platform, made it possible to screen for all reactions simultaneously by LC–MS analysis. While screening 1748 samples by LC–MS are manageable, ensuring a minimum of sample redundancy is still paramount.

## Conclusions

UGT76G1 is a chameleon enzyme catalyzing at least eight different glucosylation steps in the metabolic glycosylation grid of steviol glycosides. Several of the UGT76G1 catalyzed steps result in formation of undesired steviol glucosides (1,3-bioside, Reb G, Reb Q and Reb I), none of which can be converted to the premium steviol glucosides Reb D and Reb M (Fig. 1) [7].

Here we demonstrate how homology models of a highly promiscuous UGT can be used in combination with semi high-throughput analytic methods to effectively probe the immense sequence search space. This allowed for the identification and subsequent characterization of key residues involved in UGT specificity yielding variants, such as UGT76G1_Thr146Gly_ and UGT76G1_His155Leu_, which results in *S. cerevisiae* strains with increased or specific production of Reb D and Reb M. This demonstrated that site-saturation mutagenesis targeting the binding pocket of UGT76G1 constitutes an important tool that allows for tailored production of Reb D and Reb M. This can be used to achieve steviol glucoside products with low sweet threshold values and increased sweetness intensity.

This study is to our knowledge the most comprehensive analysis of the substrate specificity of a UGT published to date, and shows how homology modelling in combination with a yeast production strain and LC–MS make it possible to engineer a highly promiscuous enzyme towards enabling next generation stevia sweeteners.

## Additional files

**Additional file 1: Figure S1.** Product formation following incubation of *S. cerevisiae* lysate containing UGT76G1 with 1,2-stevioside or Reb A and UDPG. A novel steviol glucoside with an *m*/*z* value corresponding to steviol attached to 5 glucose moieties (*m*/*z* = 1151.150–1151.650) appeared at retention time 1.97 min which partially co-elutes with 1,2-stevioside and rebaudioside A fragment ions at 2.05–2.08 min. Based on the 1,3-glucosylation reactions catalyzed by UGT76G1, the compound is expected to be rebaudioside I [12]. NL: Noise Level.

**Additional file 2: Figure S2.** Reb D and Reb M accumulation in *S. cerevisiae* strains transformed with the *UGT76G1* site-saturation library. The shaded area indicates the Reb D/M ratio for the wild-type controls, while the dashed boxes indicate which colonies were selected for retest and sequencing of the expressed site-saturation UGT76G1 variants.

## Abbreviations

13-SMG: steviol-*C*_13_-(mono)-glucoside
19-SMG: steviol-*C*_19_-(mono)-glucoside
CH_3_CN: acetonitrile
AUC: area under the curve
epPCR: error prone PCR
ESI: electrospray ionization
DMSO: dimethyl sulfoxide
HCOOH: formic acid
ID: sequence identity
LC–MS: liquid chromatography mass spectrometry
NL: noise level
Reb A: rebaudioside A
Reb C: rebaudioside C
Reb D: rebaudioside D
Reb I: rebaudioside I
Reb M: rebaudioside M
Reb Q: rebaudioside Q
SC-ura: synthetic complete media without uracil
SIM: single ion monitoring
Tris: tris(hydroxymethyl)aminomethane
PSPG: plant secondary product glycosyltransferase
UDPG: uridine diphosphate glucose
UDP: uridine diphosphate
UDP-2FGlc: UDP-2-deoxo-2-fluoro glucose
UGT: UDPG-dependent glucosyltransferase
WT: wild-type
