# Supplementary figures and images for: Microbial production of next-generation stevia sweeteners
Source: Microb Cell Fact. 2016 Dec 7;15:207. doi: 10.1186/s12934-016-0609-1 (PMC5142139; doi:10.1186/s12934-016-0609-1)

Figure S1


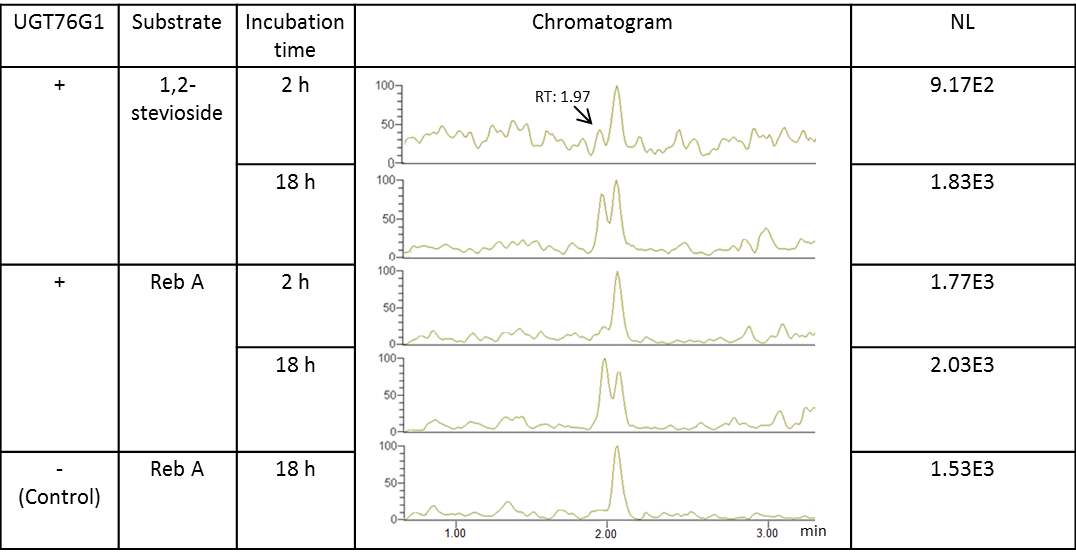

Supplement: Supplementary file 1 — Additional file 1: Figure S1. Product formation following incubation of S. cerevisiae lysate containing UGT76G1 with 1,2-stevioside or Reb A and UDPG. A novel steviol glucoside with an m/z value corresponding to steviol attached to 5 glucose moieties (m/z = 1151.150–1151.650) appeared at retention time 1.97 min which partially co-elutes with 1,2-stevioside and rebaudioside A fragment ions at 2.05–2.08 min. Based on the 1,3-glucosylation reactions catalyzed by UGT76G1, the compound is expected to be rebaudioside I [12]. NL: Noise Level. [file 12934_2016_609_MOESM1_ESM.docx]

Figure S2


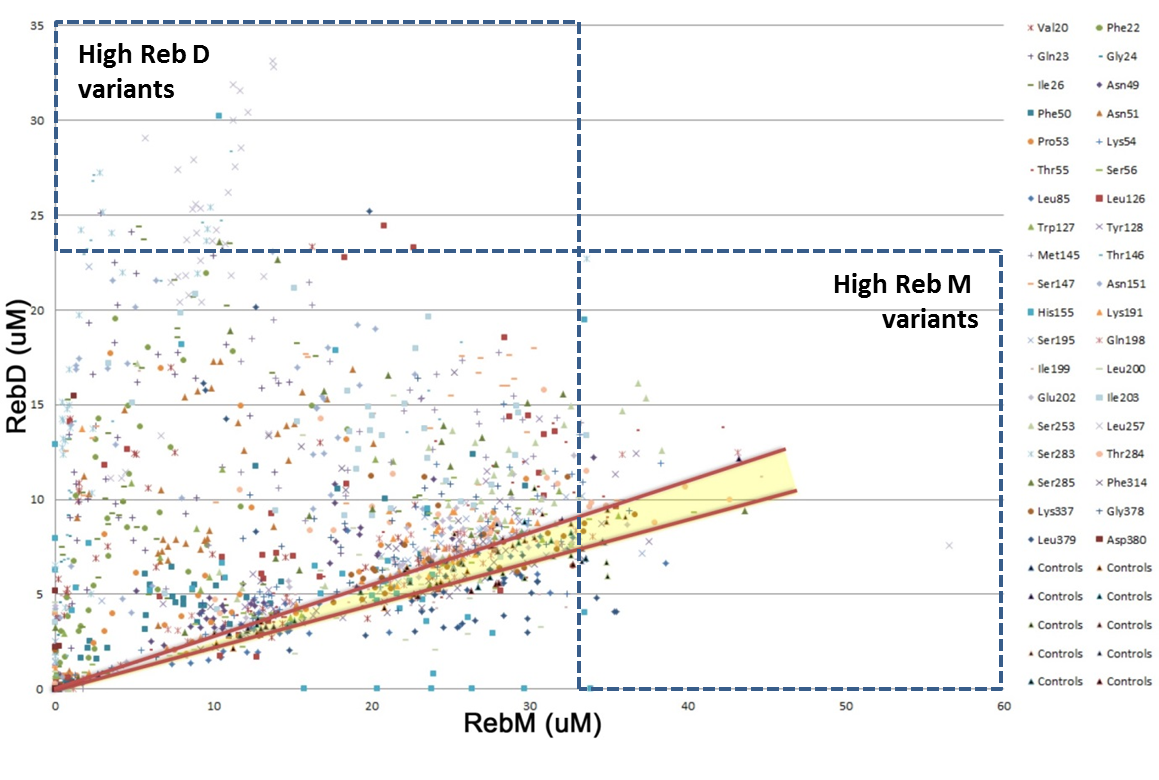

Supplement: Supplementary file 2 — Additional file 2: Figure S2. Reb D and Reb M accumulation in S. cerevisiae strains transformed with the UGT76G1 site-saturation library. The shaded area indicates the Reb D/M ratio for the wild-type controls, while the dashed boxes indicate which colonies were selected for retest and sequencing of the expressed site-saturation UGT76G1 variants. [file 12934_2016_609_MOESM2_ESM.docx]
